# Supplementary material for: Transcriptome-Assisted SNP Marker Discovery for Phytophthora infestans Resistance in Solanum lycopersicum L
Source: Int J Mol Sci. 2023 Apr 5;24(7):6798. doi: 10.3390/ijms24076798 (PMC10095378; doi:10.3390/ijms24076798)
Supplement: Supplementary file 1 [file ijms-24-06798-s001.zip › ijms-2303444-supplementary.pdf]

Tableble S1. Mapping percentage of control and infected samples to *Phytophthora infestans* genome (NCBI genome ID- ASM14294v1).

**Sample**	**Percentage of reads mapping to *Phytophthora infestans* genome**
Control Resistant 1	0.66%
Control Resistant 2	0.30%
Control Susceptible 1	0.43%
Control Susceptible 2	0.19%
Infected Resistant 1	0.75%
Infected Resistant 2	0.35%
Infected Susceptible 1	7.05%
Infected Susceptible 2	5.37%

Table S2. Summary of screened SNPs across all public domain dataset. ‘*’ shows the presence of alternative SNP allele.

**Chromosome**	**Location**	**Stupice**	**Brandywine Red**	**Matt’ s Wild Cherry**	**Prudens Purple**	**Legend**	**Cherry Roma**	**Green Zebra**	**Mr. Stripey**	**Lemon Drop**	**Mexico Midget**	**NC1-CELBR**	**LA2093**	**LA1673**	**San Marzano**	**Castle rock**	**Money Maker**	**LA4084**	Total frequency in resistant (13)	Total frequency in susceptible (4)	Gene ID (SL3.0 Refseq)
NC_015438.3	303295	*		*	*	*					*	*		*					7	0	LOC101251912
NC_015446.3	72501667	*			*						*	*	*	*					6	0	LOC101055591
NC_015446.3	72500390	*									*	*	*	*					5	0	LOC101055591
NC_015446.3	72501893	*									*	*	*	*					5	0	LOC101055591
NC_015446.3	72502022	*									*	*	*	*					5	0	LOC101055591
NC_015446.3	72502108	*									*	*	*	*					5	0	LOC101055591
NC_015446.3	72503137	*									*	*	*	*					5	0	LOC101055591
NC_015446.3	71869913	*						*				*							4	0	LOC101055591
NC_015446.3	72502973	*									*	*		*					4	0	LOC101055591
NC_015446.3	11276260									*	*		*	*					4	0	LOC101259360
NC_015446.3	71737403	*									*	*							3	0	LOC101267227
NC_015446.3	72006954	*									*	*							3	0	LOC101249573
NC_015446.3	72461464	*									*	*							3	0	LOC101263749
NC_015446.3	72462159	*									*	*							3	0	LOC101263749
NC_015446.3	72672774	*									*	*							3	0	LOC101257900

NC_015446.3	72674213	*									*	*							3	0	LOC101257900
NC_015446.3	72499807	*									*	*							3	0	LOC101055591
NC_015446.3	72674213	*									*	*							3	0	LOC101257900
NC_015444.3	1496621											*		*					2	0	LOC100191137
NC_015444.3	1927318	*			*														2	0	LOC101251003
NC_015445.3	54359369							*						*					2	0	LOC101267596
NC_015445.3	54359478							*						*					2	0	LOC101268596
NC_015445.3	54359866							*						*					2	0	LOC101268596
NC_015446.3	72503654		*									*							2	0	LOC101055591
NC_015445.3	54359647													*					1	0	LOC101267596
NC_015445.3	54359914							*											1	0	LOC101268596
NC_015446.3	72503161											*							1	0	LOC101055591
NC_015446.3	11275175													*					1	0	LOC101259360
NC_015438.3	679178																		0	0	LOC101264357
NC_015441.3	55622649		*	*		*	*			*					*				5	1	LOC101256377
NC_015438.3	74936467	*			*					*		*					*		4	1	LOC101253123
NC_015446.3	72674154	*					*				*	*						*	4	1	LOC101257900
NC_015446.3	71869964	*									*	*						*	3	1	LOC101244905
NC_015446.3	71979227	*									*	*						*	3	1	LOC101247517
NC_015446.3	72291083	*									*	*						*	3	1	LOC544097
NC_015446.3	72461703	*									*	*						*	3	1	LOC101263749
NC_015446.3	72674063	*									*	*						*	3	1	LOC101257900
NC_015446.3	72674154	*									*	*						*	3	1	LOC101257900
NC_015446.3	72856032										*	*						*	2	1	LOC101254426
NC_015446.3	72674063	*									*	*						*	2	1	LOC101257900
NC_015443.3	49157464																	*	0	1	LOC543763
NC_015447.3	64060576											*							0	1	LOC101253039
NC_015447.3	65486858	*																	0	1	LOC101249777
NC_015441.3	55117425	*		*	*	*	*								*		*		5	2	LOC101267761
NC_015449.3	65790093	*	*		*										*			*	3	2	LOC101249717

Table S3. SNPs validated with rhAmp assays and their sequences.

SNP ID	Chromosome (SL3.0)	Position	Sequence [Reference Allele/Alternate Allele]
774	NC_015446.3/Chromosome 9	72672774	TAGGTTATCCACCAGAAGGTTACTCTAAAGATGCATA[C/T]CCACCACCAGGGTATCCTCAGCAAGGGTATCC
893	NC_015446.3/Chromosome 9	72501893	AGAGTCAATCGCAACTTCCGCTTCATATGCCCAATTTC[G/T]CTTAAAATGGATGATAAAGATGACAGATCCTT
667	NC_015446.3/Chromosome 9	72501667	GATATGTCGAGTTGGGAAGTATTCAAATGGTGCTACCA[T/C]ATAGCAAGGCTTAAGAGCAATGTCCGCTGAC
390	NC_015446.3/Chromosome 9	72500390	GTCAAAATCAAGACCTTAAGAAGTCGCAATTTAAGAAT[C/T]TGACAGAAGGGAATCCCATCAATTAACTCTA
973	NC_015446.3/Chromosome 9	72502973	TCCAACACAATCCATATATTTCGACAGATCAGAATCATT[C/G]AACTCATGGTTGTAACTTAAACTGATTTTACC
22	NC_015446.3/Chromosome 9	72502022	ATCAGGAAATCTAAACAAGATTTAGATTTCGACATCTC[T/C]TTCAGTTTCCGCATAAGAGAATCCAGAAAGCT
807	NC_015446.3/Chromosome 9	72499807	ACAAAATTGGAGATACTCCAGGCTTGGTAGTCCTGCAA[T/C]GAAGGAACTCAGGTTTTCGATATGAGCCCCAC
108	NC_015446.3/Chromosome 9	72502108	TGGGGAATTGCGATGGAGTGAATTTTAAGGATTTATAGT[G/A]CGTCTCCACTTGTGCCTTCAGATCTTTAGTTTT
